# Neuroprotective Effect of Inhaled Nitric Oxide on Excitotoxic-Induced Brain Damage in Neonatal Rat

**DOI:** 10.1371/journal.pone.0010916

**Published:** 2010-06-01

**Authors:** Julien Pansiot, Gauthier Loron, Paul Olivier, Romain Fontaine, Christiane Charriaut-Marlangue, Jean-Christophe Mercier, Pierre Gressens, Olivier Baud

**Affiliations:** 1 INSERM, Hôpital Robert Debré, Paris, France; 2 INSERM, UMR 676, Hôpital Robert Debré, Paris, France; 3 INSERM UMR 711, Université Pierre et Marie Curie, Faculté de Médecine, Hôpital de la Salpêtrière, Paris, France; 4 APHP, Emergency Department, Hôpital Robert Debré, Paris, France; 5 APHP, Neonatal Intensive Care Unit, Hôpital Robert Debré, Paris, France; 6 PremUP Foundation, Paris, France; Chiba University Center for Forensic Mental Health, Japan

## Abstract

**Background:**

Inhaled nitric oxide (iNO) is one of the most promising therapies used in neonates. However, little information is known about its impact on the developing brain submitted to excitotoxic challenge.

**Methodology/Principal Findings:**

We investigated here the effect of iNO in a neonatal model of excitotoxic brain lesions. Rat pups and their dams were placed in a chamber containing 20 ppm NO during the first week of life. At postnatal day (P)5, rat pups were submitted to intracranial injection of glutamate agonists. At P10, rat pups exposed to iNO exhibited a significant decrease of lesion size in both the white matter and cortical plate compared to controls. Microglia activation and astrogliosis were found significantly decreased in NO-exposed animals. This neuroprotective effect was associated with a significant decrease of several glutamate receptor subunits expression at P5. iNO was associated with an early (P1) downregulation of pCREB/pAkt expression and induced an increase in pAkt protein concentration in response to excitotoxic challenge (P7).

**Conclusion:**

This study is the first describe and investigate the neuroprotective effect of iNO in neonatal excitotoxic-induced brain damage. This effect may be mediated through CREB pathway and subsequent modulation of glutamate receptor subunits expression.

## Introduction

Brain injury in the premature infant is a problem of major importance. Approximately 10 percent of the survivors from very preterm birth later exhibit cerebral palsy (CP) and an additional 25 to 50 percent exhibit cognitive, attentional, and/or behavioral deficits. These neurologic disabilities observed relate in considerable part to cerebral white matter injury [1]. Factors that seem involved in the pathophysiology of CP in these models include hypoxia and ischemia, infection and inflammation, excitotoxicity, accumulation of reactive oxygen species, and deficiencies in growth factors [2], [3]. These factors seem to act in combination to cause damage to the developing white matter.

Glutamate accumulation may be a mechanism common to many risk factors for CP. Glutamate, the major excitatory neurotransmitter, acts via several groups of receptors, namely, N-methyl-D-aspartate (NMDA) receptors, alpha-3-amino-hydroxy-5-methyl-4-isoxazole (AMPA) receptors, kainate receptors, and metabotropic receptors (mGluRs). Excessive activation of glutamate receptors may cause cell vulnerability, in part as a result of intracellular calcium influx [4], [5]. Intracerebral injection of glutamate agonists into the neocortex and white matter of newborn rodents produces histological lesions that mimic the brain damage observed in preterm neonates [6]–[8]. In addition to excitotoxicity, nitric oxide is recognized as being a key modulator of risk factors involved in CP, by regulating vascular tone, reperfusion, inflammation and oxidative stress [9], [10].

Despite considerable advances in our understanding of the pathophysiology of brain damage during development, therapeutic options are still extremely limited. Inhaled nitric oxide (iNO) is one of the most commonly used therapies, promising but also controversial, in neonatal intensive care units. This molecule is thought to have only a local effect, limited to the vascular tone of the lungs, and has been proposed to treat pulmonary hypertension-related hypoxemia and chronic lung disease. However, increasing experimental and clinical evidences suggest that iNO could also have an impact on the developing central nervous system [11], [12].

Here, we describe the neuroprotective effect of iNO in neonatal excitotoxic-induced brain damage. This effect appears to be mediated through pAkt-pCREB pathway and subsequent modulation of glutamate receptor subunits expression.

## Materials and Methods

### Ethics statement

Full details of the study have been approved by Robert Debré research council review board; the approval number is 2009-02. All experiments were carried out in compliance with the ethical rules of INSERM.

### Animals and model of excitotoxic brain lesions

Twenty-four hours before delivery, pregnant female rats (Sprague-Dawley, Janvier, Le Genest-St-Isle, France) were placed in a normoxic, normocapnic gas chamber containing either 5 or 20 ppm inhaled NO and <1 ppm NO_2_ for postnatal days (P) 0 to 7. The concentration of NO and NO_2_ was monitored using the iNOvent system (INOTherapeutics, Clinton, NJ).

P5 rat pups of both sexes were used for this study. Ibotenate (Tocris, Bristol, UK), NMDA (Tocris) and S-Willardiine (Tocris) were diluted in phosphate buffer saline (PBS). Ibotenate activates NMDA and metabotropic glutamatergic receptors while S-Willardiine activates both AMPA and kainate receptors. Ibotenate (10 µg), NMDA (4 µg) or S-Willardiine (15 µg) was injected intracerebrally on P5 to rat pups, as previously described [6]. Briefly, rat pups anesthetized with isoflurane were kept under a warming lamp to maintain body temperature. They were injected intracerebrally (into the neopallial parenchyma) on P5. Intracerebral injections were performed with a 26-gauge needle on a 50 µl Hamilton syringe mounted on a calibrated microdispenser. The needle was inserted 2 mm below the external surface of skin. The tip of the needle was placed in the frontoparietal area of the right hemisphere, 2.5 mm from the midline in the lateral-medial plane, and 4 mm from the bregma in the rostro-caudal plane. It was confirmed by histopathological observation that the tip of the needle always reached the periventricular white matter. Two 1 µl boluses of ibotenate, NMDA or S-Willardiine were injected at 20 second intervals. The needle was left in place for an additional 20 seconds.

In a first set of experiments, P5 rat pups were intracerebrally injected with PBS. Pups treated with intracerebral PBS injections had minimal lesions, mostly consisting of needle tracks, as previously reported [13]. Therefore, control animals were kept into room air and treated animals were exposed to iNO.

At least 12 animals of each treatment group were killed by decapitation 5 days after the injection, and the brains were processed as previously described [14]. In all the experiments described below, two investigators blinded to treatment group determined the size of the lesion in each pup.

### Determination of lesion size

Rat pups were sacrificed by decapitation 5 (P10) or 25 (P30) days after the excitotoxic challenge. Brains were fixed immediately in 4% formalin and remained in this solution for 5 days. Following paraffin embedding, we cut 16-µm thick coronal sections. Every third section was stained with cresyl-violet. The size of neocortical and white matter lesions can be defined by the length on three orthogonal axes: the lateral-medial axis (in a coronal plane), the radial axis (also in a coronal plane, from the pial surface to the lateral ventricle), and the fronto-occipital axis (in a sagittal plane). In previous studies [6], [14], we found an excellent correlation among the measurements from the three axes of the excitotoxic lesions. Based on these findings, we cut serial sections of the entire brain in the coronal plane for this study. This permitted an accurate and reproducible determination of the sagittal fronto-occipital diameter (which is equal to the number of sections where the lesion was present multiplied by 16 µm). We used this measure as an index of the lesion volume.

### Immunohistochemistry

In each experimental group, we studied 6 to 10 pups in three separate experiments. Immunolabeling with the primary antibodies listed in File S1 was visualized using the streptavidin-biotin-peroxidase method as previously described [15].

Immunoreactive cells were counted in the white matter underlying the cortex (+2.16 to −0.36 mm from the bregma) in animals sacrificed on P10. Cells were counted within a 0.25 mm^2^ grid, in at least 4 sections per animal, and 6 or more animals per group.

### Optical density of pCREB-positive cells

The optical density of pCREB-immunoreactive cells was measured in the cortical plate in coronal sections (+2.16 to −0.36 mm from the bregma). At least 4 sections each from 6 to 10 animals per group, sacrificed on either P1 or P7, were examined. Optical density was measured at 20× magnification using a computerized image analysis system (ImageJ, NIH, MA, http://rsb.info.nih.gov/ij/). Nonspecific background density was measured at each brain level in an area devoid of pCREB immunostaining, and subtracted from the values for the cortex.

### Quantitative real-time PCR

DNA-free total RNA from the brain cortex including the white matter was obtained using a protocol adapted from Chomczynski and Sacchi [16]. Quantitative RT-PCR was used to assess gene expression for VEGF, VEGF-R1 and 2 and for glutamate receptor subunits GluR1–4, NR1, NR2A, NR2B, NR2C and NR2D and mGluR1–8. To standardize gene expression across samples, we first compared the expression levels of four well-known housekeeping genes within the samples. For reverse transcription, we used 600 ng of total RNA and the Iscript cDNA synthesis kit (Bio-Rad, Hercules, CA). Real-time PCR was set up with Supermix (Bio-Rad) containing syber green for 50 cycles with a three-step program. Each reaction was run twice with a least 6 animals per group, and in both cases, samples were assessed in triplicate. The applied primers for real time-PCR have been previously reported [17].

### Western Blot

Membrane proteins were extracted from forebrain cortex, including white matter, taken in P1 and P7 rat pups. Extraction was achieved by homogenization in Hepes buffer containing protease inhibitors from Sigma, according to the manufacturer's instructions. We loaded 50 µg of protein from each sample were incubated overnight with either a Akt, pAkt, pCREB, ERK1/2 or pERK1/2 antibody or an anti-α-actin antibody. Western blot experiments were run in triplicate with at least 4–6 animals (see File S1).

### ELISA assays

We used specific ELISA assays from R&D system Europe (Lille, France) to quantitate VEGF and cAMP Response Element Binding Protein (CREB) phosphorylated at S133 levels in the whole brain samples according to the manufacturer's instructions.

### Statistical analysis

All data were reported as means ± S.E.M. Analysis of variance was performed with age and groups as the factors, and the Newman-Keuls post-hoc test was used. Statistical tests were run on GraphPad Prism version 4.00 (GraphPad Software, San Diego, CA).

## Results

### Effect of iNO on brain lesion induced by intracranial injection of various glutamate agonists

**Figure 1:**
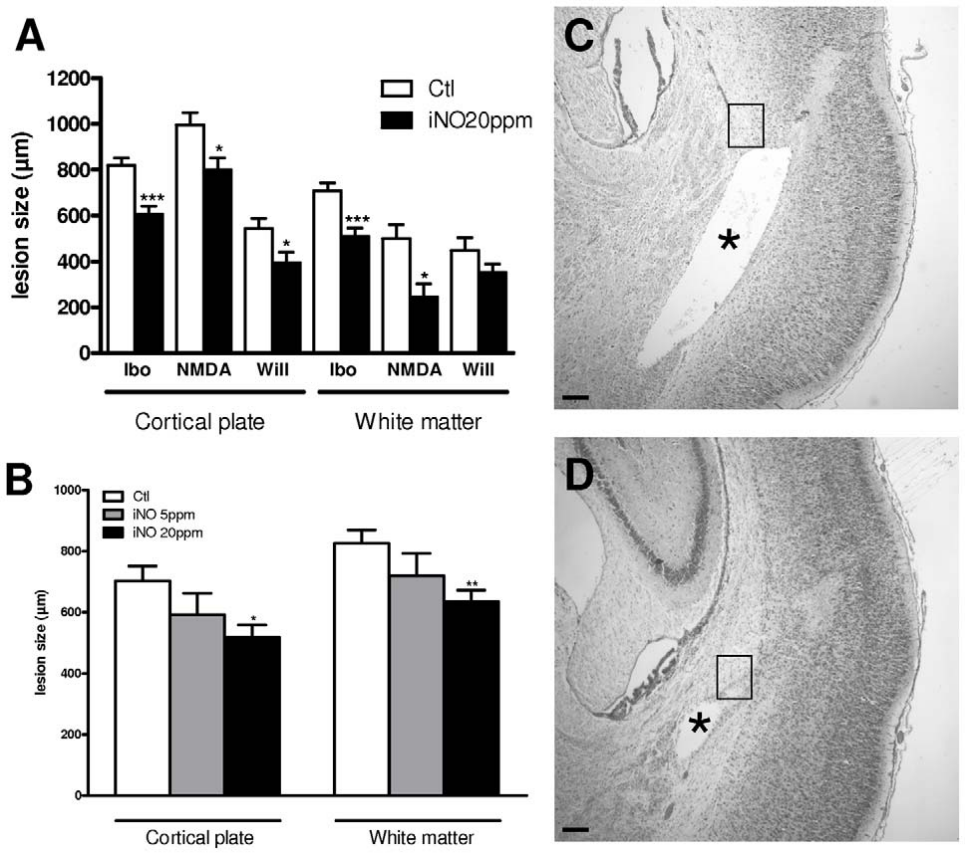
iNO confers neuroprotection in excitotoxic-induced brain lesion in neonatal rat. A. Quantitative analysis of lesion size in cortical plate and white matter induced by ic injection of either ibotenate (ibo), NMDA or S-willardiine (will) in P5 rat pups with and without iNO exposure (***p<0.001; *p<0.05). B. Dose-dependence of the neuroprotective effect of iNO at 5 and 20 ppm in rat pups subjected to ic injection of ibotenate (**p<0.01; *p<0.05). C–D. Cresyl violet–stained sections showing brain lesions induced by ibotenate intracerebrally injected on P5 and studied at the age of P10. iNO exposure reduced ibotenate-induced cortical lesion and white matter cyst (asterisk). Bar = 50 µm. Boxes delineate the regions in which glial cell counts were performed.

Rat pups injected on P5 with ic ibotenate, NMDA or S*-*Willardiine developed cortical lesions and periventricular white matter cysts in all cases. The cortical lesion was typically characterized by neuronal loss in all neocortical layers and almost complete disappearance of neuronal cell bodies along the axis of excitotoxin injection. In P5 rats, iNO 20 ppm induced a significant neuroprotection of both the cortical plate and the developing white matter against ibotenate- or NMDA-induced lesions (Figure 1A, C–D); in contrast, iNO induced a moderate neuroprotection of the cortical plate but not white matter of S*-*Willardiine-induced brain lesions when observed on P10 (Figure 1A). The protective effect of iNO was dose-dependent as iNO at 5 ppm induced a non significant trend to decrease the cortical and white matter lesion size in excitotoxic-induced lesions (Figure 1B). No significant neuroprotective effect was detected when rat pups were exposed to iNO only after the excitotoxic challenge. No difference was observed between male and female pups.

**Figure 2:**
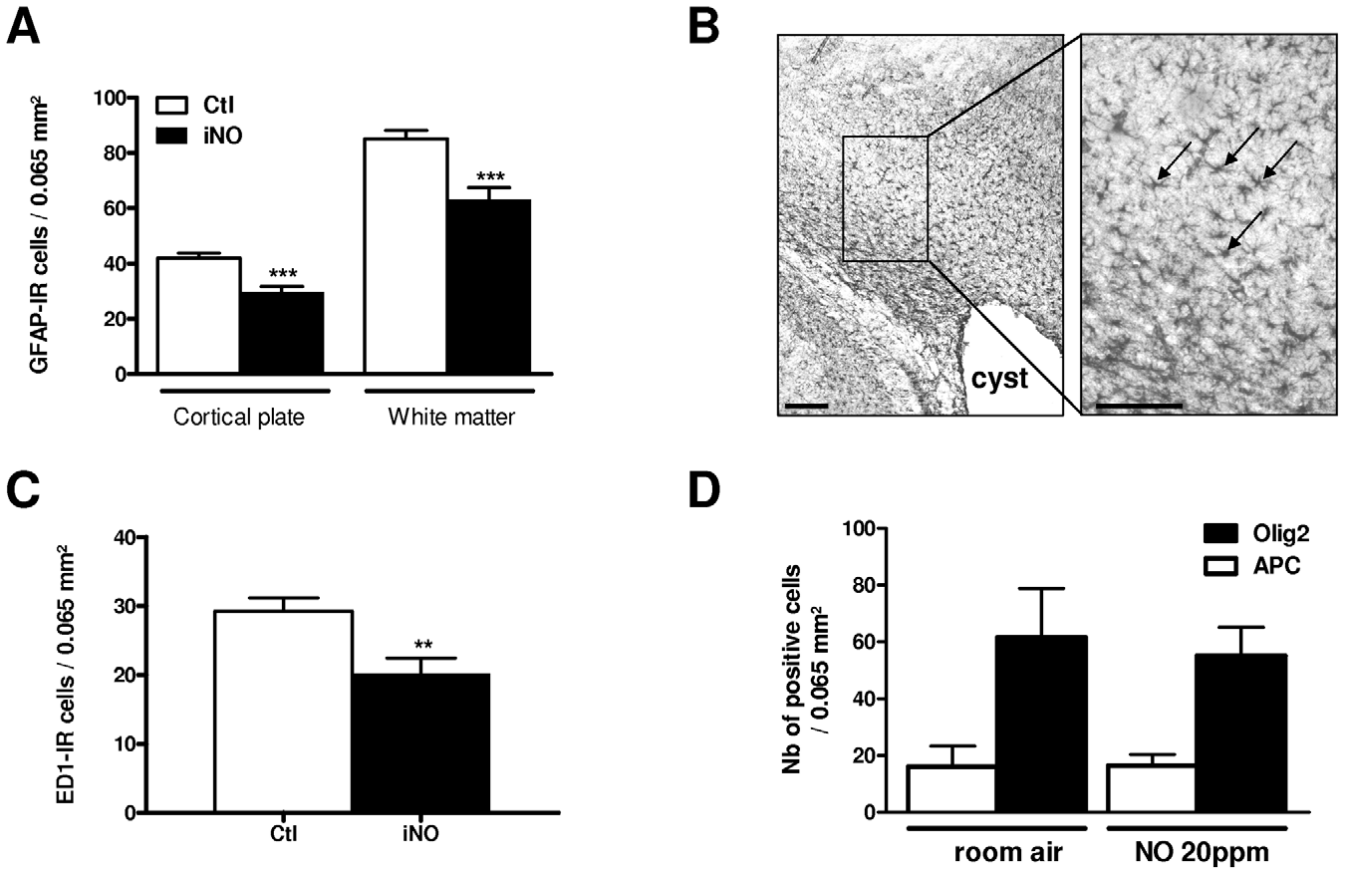
iNO reduces glial reaction around excitotoxic-induced brain lesion in neonatal rat. A: Quantitative analysis of GFAP-positive cells density in P10 rat pups with and without iNO exposure in cortical plate and white matter (***p<0.001). B: Coronal sections showing GFAP+ cells within white matter in room air-exposed P10 rat pups (controls). Activated mature astrocytes displayed number of processes and enlarged cell bodies (arrows). Bars = 50 µm. C: Quantitative analysis of ED1-positive cells density in P10 rat pups with and without iNO exposure around the white matter lesion (**p<0.01). D: Quantitative analysis of Olig2- and APC-positive cells density in P10 rat pups with and without iNO exposure around the white matter lesion.

Because maximum neuroprotective effect was observed when ibotenate injection was associated with iNO 20 ppm, further experiments were conducted using this excitotoxin and iNO concentration. In the developing white matter, iNO 20 ppm reduced ibotenate-induced astrogliosis (GFAP) and microglial activation (ED-1) when analyzed 5 days after the insult. Glial cells counts were performed within external capsule around white matter cyst (see Figure 1C). To analyze cell types in the glial scars in the injured white matter, mature activated astrocytes density was assessed by counting GFAP-positive cells with number of processes and enlarged cell bodies in P10 rat pups (Figure. 2 A–B). This density was found significantly higher in pups exposed to room air compared to those exposed to iNO. Because ic injection of excitotoxins was associated with infiltration of monocytes-macrophages around white matter lesion, we measured microglial cells density using ED-1, a marker for macrophages [13]. Activated microglial cells density was found significantly lower in iNO-treated animals (Figure 2C). When compared to control animals, iNO failed to increase the density of total population of oligodendrocytes around the white matter lesion; similarly, iNO was unable to improve the density of cells labelled with APC, a marker of mature oligodendrocytes (Figure 2D).

### Effect of iNO on glutamate receptors subunits gene expression

**Figure 3:**
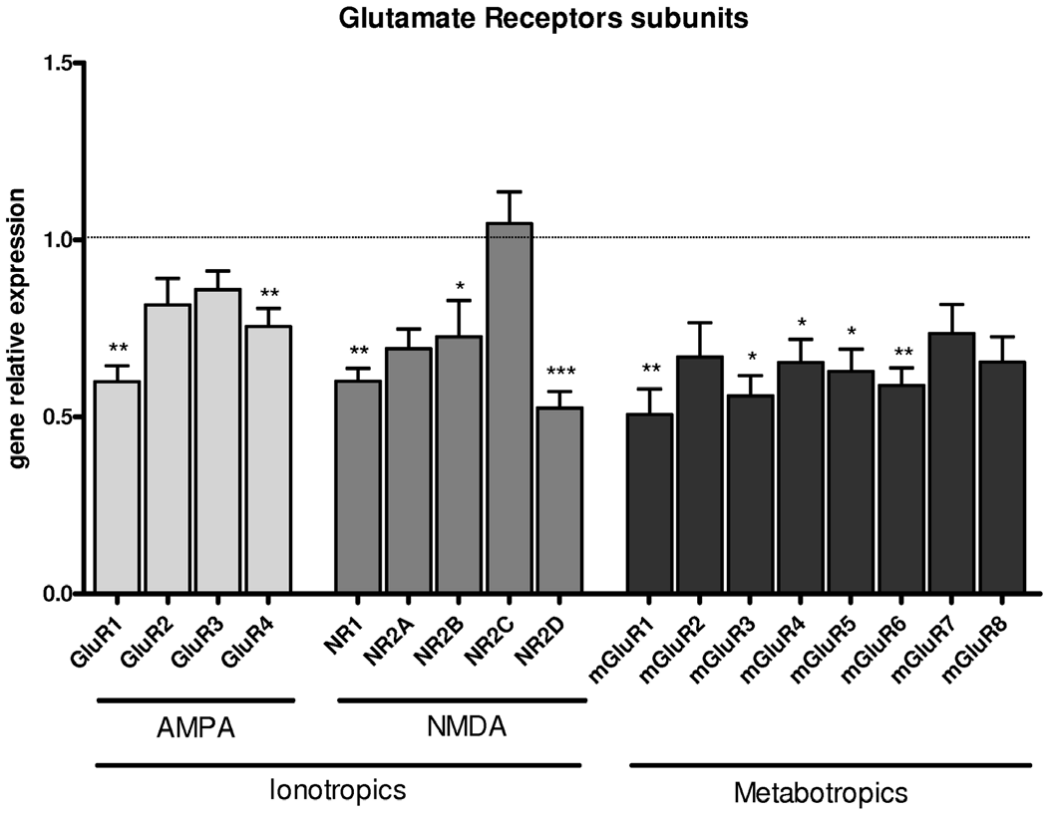
iNO dowregulates several glutamate receptor subunits gene expression. Relative gene expression of glutamate receptor subunits in P5 rat pups subjected to 20 ppm iNO normalized to controls (***<0.001; **p<0.01; *p<0.05).

Because excitotoxicity and genetic regulation of glutamate-receptor expression are known to play a key role in brain damage [17], we investigated whether glutamate-receptor gene expression was altered by iNO, and may account for the neuroprotective effect of iNO. We assessed the expression of glutamate-receptor subtypes using quantitative real-time PCR demonstrating a downregulation of most of them just before the intracranial injection of glutamatergic agonist in P5 rat pups. Significant 1.5 to 2-fold downregulation was observed for AMPA/kaïnate-receptor subunits GluR1 and 4, the NMDA receptor subtypes NR1, 2B and 2D, and the metabotropic receptor subunits mGluR1, 3, 4, 5, 6, 7 in at least 6 animals in two separate experiments, compared to normoxic controls (Figure 3). Again, no difference was observed between male and female pups.

### Signaling pathway involved in the neuroprotective effect of iNO on excitotoxic-induced brain lesion

**Figure 4:**
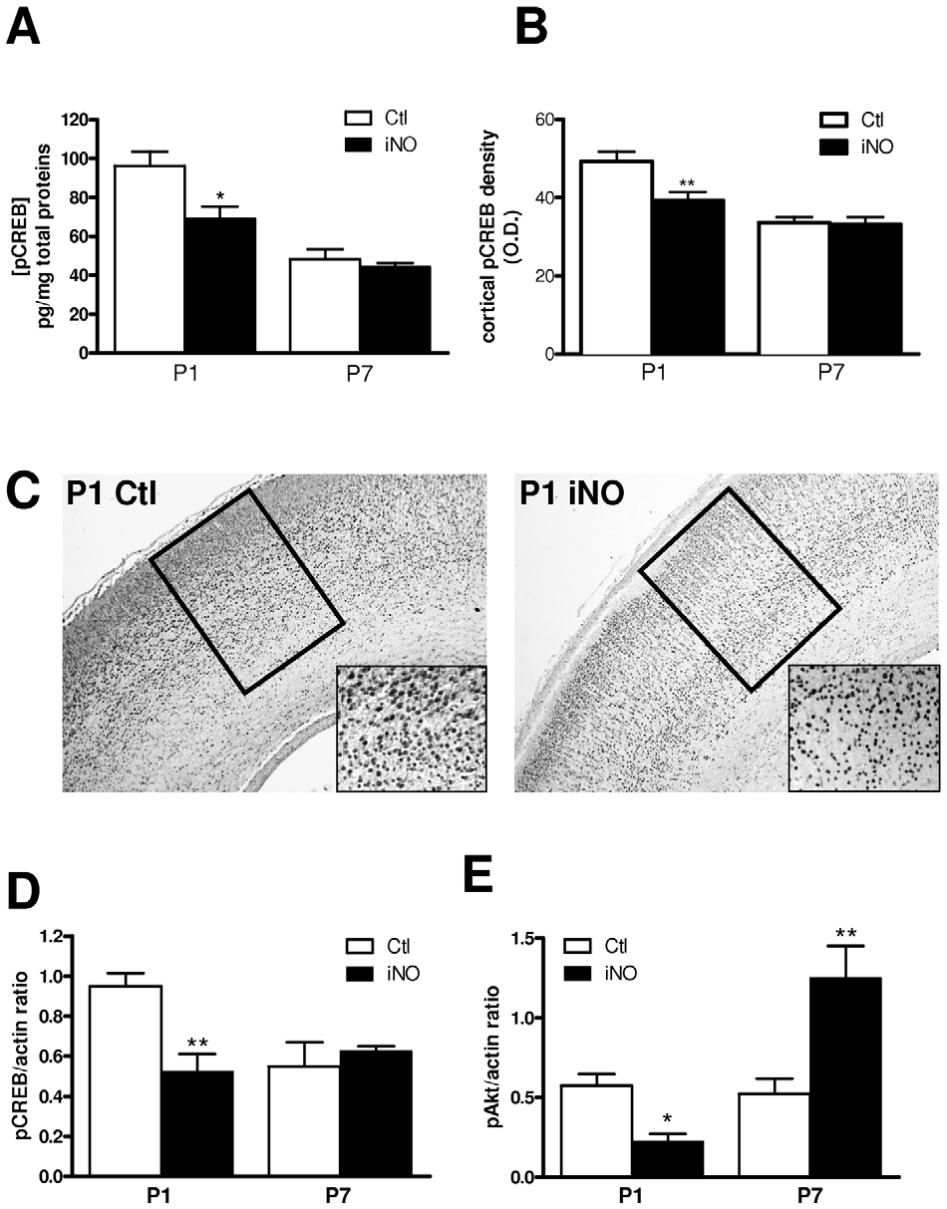
pAkt-pCREB pathway is involved in the neuroprotective effect of iNO. A. Quantitative analysis of pCREB protein content of P1 and P7 brain measured by ELISA assay (*p<0.05). B. Quantitative analysis of cortical pCREB immunoreactivity optical density in P1 and P7 rat pups subjected to iNO compared to controls (**p<0.01). C. Photomicrographs (with enlarged image) showing nuclear localization of cortical pCREB immunostaining in P1 rat pups with (iNO) or without (Ctl) NO (20 ppm) exposure. The area of optic density measurement (B) is indicated by a square. D, E. Quantitative analyses of western blot using pCREB (D) and pAkt (E) in response to iNO exposure (20 ppm) in P1 (before excitotoxic challenge) and P7 (after excitotoxic challenge) rat pups (*p<0.05; **p<0.01).

Finally, we explored a potential signaling pathway acting as a common modulator of glutamate-receptor expression in response to iNO. We hypothesized that CREB/Akt signaling pathway might be involved as CREB is recognized to bind the CRE sequence in the promoter of several glutamate receptor genes [18]. First, we found a significant reduction of pCREB protein concentration 24 h after the onset of NO exposure in P1 but not in P7 rat pups. This decrease was observed on ELISA assays, immunocytochemistry and western blot as well as (Figure 4A–D). To delineate upstream signaling pathways we further explore VEGF-Akt and ERK expression in response to NO exposure. We found that iNO was associated with a significant decrease in brain VEGF concentration (ELISA) and gene expression (but no change in VEGFR1 and VEGFR2 using qPCR). Furthermore, iNO induced a decrease in Akt and phosphorylated Akt in P1 rat pups (Figure 4E). In contrast, Erk1/2 expression was found similar in iNO-exposed animals compared to controls (data not shown). In contrast to these early effects of iNO on pAkt/pCREB expression observed at P1, we found that iNO did not change pCREB protein level and induced a significant increased of pAkt protein concentration in P7 brains in response to excitotoxic challenge (Figure 4E). Therefore, we speculate that complex modulation of pAkt-pCREB signaling pathway by exogenous NO could be involved in its impact on glutamate receptors regulation and excitotoxic brain damage.

## Discussion

We demonstrated here that iNO exposure during the first week of postnatal life significantly reduced the lesion size in an excitotoxic-induced brain lesions rat model. This effect appears to be associated with early downregulation of pCREB expression and subsequent downregulation of several glutamate receptors subunits.

It is now well established that NO is a physiological mediator of the central nervous system. The role of NO in developing brain remains poorly understood, but it seems to be involved in the regulation of cerebral blood flow, and in memory acquisition. In fact, NO appears to be a double-edged sword, simultaneously neurotoxic and neuroprotective. Numerous experimental studies demonstrated the deleterious effects of nitrogen reactive species accumulation in ischemic-reperfusion cerebral injury through depletion of energy, lipid peroxidation, protein nitrosylation, DNA alterations and increased permeability of the blood brain-barrier [19]–[21]. Hypoxia-ischaemia results in inflammation, especially in the developing white matter. High concentrations of NO and peroxynitrite produced locally by activated microglia may become toxic to neurons and immature oligodendrocytes *in vitro* [22], [23]. It is also well known that hypoxia-ischaemia results in the accumulation of extracellular glutamate, inducing the excitotoxicity cascade that causes neuronal death. More or less effective neuroprotection can be achieved by using NOS inhibitors that inhibit nNOS at the early phase and iNOS during the reperfusion of hypoxic insult [24].

In the other hand, endogenous NO could also result in contradictory effects, probably as a function of the intracellular redox state [25]. Endogenously produced NO in the brain also regulates local blood flow and could therefore offer neuroprotection [21], [26]. An increase in brain infarct volume has been reported in the sheep and rat when NO production is decreased by NOS inhibitors [27]. Transgenic mice helped dissect out the respective contribution of nNOS, as the extent of the lesions after medial cerebral artery occlusion is reduced in eNOS^−/−^ [28], whereas secondary neuronal damage-induced by prolonged ischemia is lessened in iNOS^−/−^ [29]. The protection conferred on the ischemic brain by NO seems to be linked to vasodilatation which improves cerebral blood flow and hinders capillary microthrombi formation. Thus, NO seems to be beneficial on the brain mostly through its vasodilatory effects, and maybe its potentially proangiogenic effects.

In contrast to the numerous studies focused on endogenous NO, almost no data was available on the experimental effect of exogenous inhaled NO. Extrapulmonary effect of iNO on reduction of myocardial infarction size and improved left ventricular systolic function have been shown in a murine model [30]. These studies demonstrate that an extrapulmonary vasoprotective effect of iNO is possible. There are several possible mechanisms for neuroprotection with iNO as well, such as modulation of circulating neutrophils, monocytes, and platelets as they pass through the lung [31]. iNO down-regulates lung-derived cytokines and free radicals production, which may lead to a decrease in brain injury [32]. Another possible mechanism in neuroprotection may relate to delivery of NO or NO-related metabolites, such as hemoglobin-derived S-nitrosothiol or nitrites/nitrates, which account for a distant vasodilatory activity [32], [33]. NO may also play an important role in ischemic preconditioning *in vivo* [34].

Clinically, the impact of iNO on the development of the central system remains controversial. For many years, iNO was feared to increase the incidence of intracranial haemorrhage in critically ill preterm neonates, because NO was demonstrated to increase bleeding time and inhibit platelet aggregation [35]. Later clinical studies demonstrated there was no significant increase in intracranial bleeding in preterm neonates [31]. Furthermore, both Schreiber et al. [36], and Kinsella et al. [37] found a lower incidence of severe brain damage in the iNO treated group, respectively. More intriguing and exciting was the fact that this short-term improvement translated into a significant improvement in neurodevelopmental outcome in the group given iNO at two-year follow-up, and this was primarily due to a 47% decrease in the risk of cognitive impairment [38].

It is conceivable that a downregulation of glutamate receptors could reduce the lesion size induced by glutamate agonists. Here, we found that iNO-induced neuroprotection was associated with an early downregulation of pCREB expression and downregulation of several glutamate receptors subunits. CREB activates gene transcription in response to elevation of intracellular cAMP levels which in turn phosporylates CREB at Ser133. Phosphorylated CREB binds to the cAMP response element (CREs), represented by the palindromic consensus sequence TGACGTCA found in the 5′ flanking region of target genes [18]. This consensus sequence was found in the 5′ flanking region of most of the iNO-regulated glutamate receptors genes (ie, NR1, NR2B, mGlu1, 3 and 6) but interestingly, was not found in the 5′ flanking region of NR2C gene which transcription was unchanged in iNO-exposed rat pups (see Figure 2). Mutational analysis demonstrated that transcription of NR1 and NR2B are regulated by the c-AMP signaling pathway, most likely through the binding of CREB and its activation by signal-dependent phosphorylation [18], [39]. Conversely, glutamate receptor activation and subsequent calcium influx may activate CREB shortly [40]. Moreover, most data indicated that pAkt was found neuroprotective [41]. In our model, CREB transcription level was found unchanged after the excitotoxic insult and pAkt protein level was significantly increased in response to excitotoxic challenge in P7 iNO-exposed rat pups as compared to room air-exposed controls. In addition to calmoduline-dependent kinases and MAPK/ERK kinases pathways, the accumulation of cAMP in response to G-protein-coupled receptors also induces activation of Akt/proteine kinase B which directly or indirectly affects CREB [42]. Here, our data suggest that exogenous NO induced subtle modulation of Akt/CREb signaling pathway in the developing brain. We speculate that iNO may induce an early downregulation of pCREB and subsequent upregulation of pAkt after excitotoxic insult leading to a decreased expression of several genes including those encoding for glutamate receptors subunits, and subsequent neuroprotection.

In conclusion, this study is the first to describe and to investigate the neuroprotective effect of iNO in neonatal excitotoxic-induced brain damage. This effect appears to be associated with changes in VEGF-pAkt-pCREB and glutamate receptor subunits expression. Further preclinical studies are needed to confirm the ability of iNO to induce neuroprotection in other animal models of perinatal brain damage.

## Supporting Information

File S1 Primary antibodies used for immunohistochemistry and western blot analyses.
(0.04 MB DOC)
