# Supplementary material for: Neuroprotective Effect of Inhaled Nitric Oxide on Excitotoxic-Induced Brain Damage in Neonatal Rat
Source: PLoS One. 2010 Jun 1;5(6):e10916. doi: 10.1371/journal.pone.0010916 (PMC2879374; doi:10.1371/journal.pone.0010916)
Supplement: File S1 — Primary antibodies used for immunohistochemistry and western blot analyses. (0.04 MB DOC) [file pone.0010916.s001.doc]

**File S1**

Primary antibodies used for immunohistochemistry and western blot analyses.

| **Markers** | **Labeled structures** | **Manufacturer** | **Dilution** |
| --- | --- | --- | --- |
| Olig2 | oligodendroglial lineage | IBL, Hamburg, Germany | 1/200 |
| APC | Post-mitotic oligodendrocytes | Calbiochem, La Jolla, CA, USA | 1/500 |
| ED-1 | Resident, activated microglia and blood vessels. | Vector, Burlingame, CA, USA | 1/1000 |
| GFAP | Mature astrocytes | Sigma Biosciences, St Louis, MO, USA | 1/500 |
| CREB (Phospho-Ser133) |  | Signalway Antibody, Pearland, TX, USA | 1/5000 |
| - Akt, pAkt,  - p44/42 MAPK (Erk1/2), Phospho-p44/42 MAPK (Erk1/2) (Thr202/Tyr204) |  | Cell Signaling, Danvers, MA, USA | 1/5000 |
| Alpha actin | Cytosqueleton | Santa Cruz Biotechnology, Santa Cruz, CA, USA | 1/10000 |
